# Predicting Neuroinflammation in Morphine Tolerance for Tolerance Therapy from Immunostaining Images of Rat Spinal Cord

**DOI:** 10.1371/journal.pone.0139806

**Published:** 2015-10-05

**Authors:** Shinn-Long Lin, Fang-Lin Chang, Shinn-Ying Ho, Phasit Charoenkwan, Kuan-Wei Wang, Hui-Ling Huang

**Affiliations:** 1 Department of Anesthesiology, Tri-Service General Hospital and National Defense Medical Center, Taipei, Taiwan; 2 Department of Anesthesiology, Kang-Ning General Hospital, Taipei, Taiwan; 3 Institute of Bioinformatics and Systems Biology, National Chiao Tung University, Hsinchu, Taiwan; 4 Department of Biological Science and Technology, National Chiao Tung University, Hsinchu, Taiwan; 5 Institute of Molecular Medicine and Bioengineering, National Chiao Tung University, Hsinchu, Taiwan; Xi'an Jiaotong University School of Medicine, CHINA

## Abstract

Long-term morphine treatment leads to tolerance which attenuates analgesic effect and hampers clinical utilization. Recent studies have sought to reveal the mechanism of opioid receptors and neuroinflammation by observing morphological changes of cells in the rat spinal cord. This work proposes a high-content screening (HCS) based computational method, HCS-Morph, for predicting neuroinflammation in morphine tolerance to facilitate the development of tolerance therapy using immunostaining images for astrocytes, microglia, and neurons in the spinal cord. HCS-Morph first extracts numerous HCS-based features of cellular phenotypes. Next, an inheritable bi-objective genetic algorithm is used to identify a minimal set of features by maximizing the prediction accuracy of neuroinflammation. Finally, a mathematic model using a support vector machine with the identified features is established to predict drug-treated images to assess the effects of tolerance therapy. The dataset consists of 15 saline controls (1 μl/h), 15 morphine-tolerant rats (15 μg/h), and 10 rats receiving a co-infusion of morphine (15 μg/h) and gabapentin (15 μg/h, Sigma). The three individual models of astrocytes, microglia, and neurons for predicting neuroinflammation yielded respective Jackknife test accuracies of 96.67%, 90.00%, and 86.67% on the 30 rats, and respective independent test accuracies of 100%, 90%, and 60% on the 10 co-infused rats. The experimental results suggest that neuroinflammation activity expresses more predominantly in astrocytes and microglia than in neuron cells. The set of features for predicting neuroinflammation from images of astrocytes comprises mean cell intensity, total cell area, and second-order geometric moment (relating to cell distribution), relevant to cell communication, cell extension, and cell migration, respectively. The present investigation provides the first evidence for the role of gabapentin in the attenuation of morphine tolerance from phenotypic changes of astrocytes and microglia. Based on neuroinflammation prediction, the proposed computer-aided image diagnosis system can greatly facilitate the development of tolerance therapy with anti-inflammatory drugs.

## Introduction

Morphine has long been used as a potent analgesic for pain management in clinical settings; however, long-term use results in the development of tolerance through two possible mechanisms (within-system and between-systems) [1]. Within-system tolerance is embroiled in the signal transduction of opioid receptors, comprising down-regulation of opioid receptors, functional decoupling of opioid receptors from G-proteins, and β-arrestin recruitment to opioid receptors, which results in receptor desensitization and internalization/endocytosis [2]. Moreover, N-methyl-D-aspartate acid receptors participate in the formation of opioid tolerance [3]. Between-systems tolerance is characterized by alterations in primary drug-sensitive system adaptation, including the glutamatergic receptor system [4] and glial cell activation with the release of proinflammatory cytokines [5], thus inhibiting the analgesic effect of morphine [2].

Several studies have demonstrated that suppression of neuroinflammation by inhibiting microglial activation and proinflammatory cytokines, TNF-α, IL–1β, and IL–6 is a worthwhile strategy for enhancing the anti-nociceptive effect of morphine and will inhibit the formation of morphine tolerance [5–7]. Chronic morphine treatment of astrocyte-neuron co-cultures reduces neurite outgrowth and synapse formation [8]. Midbrain astrocytes also play an important role in the development of analgesic tolerance to morphine [9]. Recent studies have sought to reveal the mechanism of opioid receptors and neuroinflammation by observing morphological changes of astrocytes and microglia. Compared to saline controls, spinal cord sections from rats administered with chronic morphine show significantly increased immune-labelling of astrocytes and microglia [10]. To evaluate the therapeutic effects of anti-inflammatory drugs and tolerance therapy, this work investigates three types of cells in the spinal cord (i.e. astrocytes, microglia, and neurons) in multiplex immunostaining images.

Co-administration of a second drug with an opioid is an effective stratagem for enhancing the antinociceptive effect and attenuating the development of tolerance [11,12]. Gabapentin is used as an anti-convulsant drug for epilepsy and is also widely accepted as an effective alternative choice for the management of neuropathic and inflammatory pain [13,14]. In animal studies, gabapentin has also been shown to have analgesic effects in several chronic pain models, including arthritic, peripheral and central neuropathic pain [15]. Although the related mechanisms have yet to be clearly understood, evidence indicates that gabapentin may act at the α2/δ-1 subunit of the calcium channel, and thus likely suppresses calcium currents and prevents extracellular calcium entry [16]. Gabapentin has also been shown to modulate several targets, including N-methyl-D-aspartate acid receptors, protein kinase C, transient receptor potential channels, and inflammatory cytokines [17].

Several preclinical studies have revealed that gabapentin contributes to an increased analgesic effect for morphine in an acute model of nociception and in a visceral nociception model [18]. However, the definite mechanism of the gabapentin augmentation of morphine-induced anti-nociceptive effect in a neuropathic pain model is not clear. Bao *et al*. found that gabapentin increases the anti-nociceptive effect of morphine through activating the expression of IL–10 and its downstream HO–1 signal pathway to suppress proinflammatory cytokine expression in a neuropathic pain model of rats [19]. Moreover, neuroinflammation counteracts the opioid’s analgesic effect.

High content screening (HCS) is known as an efficient approach to drug discovery, and can measure cellular responses to chemical disturbances in a high-throughput manner [20]. HCS provides an image-based readout of cellular phenotypes, including features such as shape, intensity, and texture in a highly multiplex and quantitative manner. Charoenkwan *et al*. proposed the HCS-Neurons method which uses HCS to identify phenotypic changes in multi-neuron images upon drug treatments [21]. The present work proposes a novel method, HCS-Morph, to predict neuroinflammation in morphine tolerance to facilitate the development of tolerance therapy based on HCS-based immunostaining images of cells in the spinal cord, including astrocytes, microglia, and neurons. We also explore potential applications of the proposed image diagnosis system to evaluate the therapeutic effect of tolerance therapy, such as the use of gabapentin for the inhibition of spinal cord neuroinflammation to restore the analgesic effect of morphine in rats.

The training dataset for modeling consists of 15 saline controls and 15 morphine-tolerant rats with neuroinflammation. In addition, 10 rats with a co-infusion of morphine and gabapentin are used to assess the therapeutic effect of gabapentin. The three individual models of astrocytes, microglia, and neurons for predicting neuroinflammation respectively yield Jackknife test accuracies of 96.67%, 90.00%, and 86.67%. The prediction results using the three models of astrocytes, microglia, and neuron cells on the 10 co-infused rats respectively show that 100%, 90%, and 60% of rats belong to the class of non-neuroinflammation. The proposed feature set for predicting neuroinflammation of astrocytes comprises the mean cell intensity, total cell area, and second-order geometric moment (relating to cell distribution), relevant to cell communication, cell extension, and cell migration. Moreover, this work proposes a novel insight into the anti-neuroinflammatory effect of gabapentin in the attenuation of morphine tolerance from phenotypic changes of astrocytes and microglia. The proposed computer-aided image diagnosis system can thus greatly facilitate the development of tolerance therapy with anti-inflammatory drugs.

## Materials and Methods

### Materials

**Fig 1:**
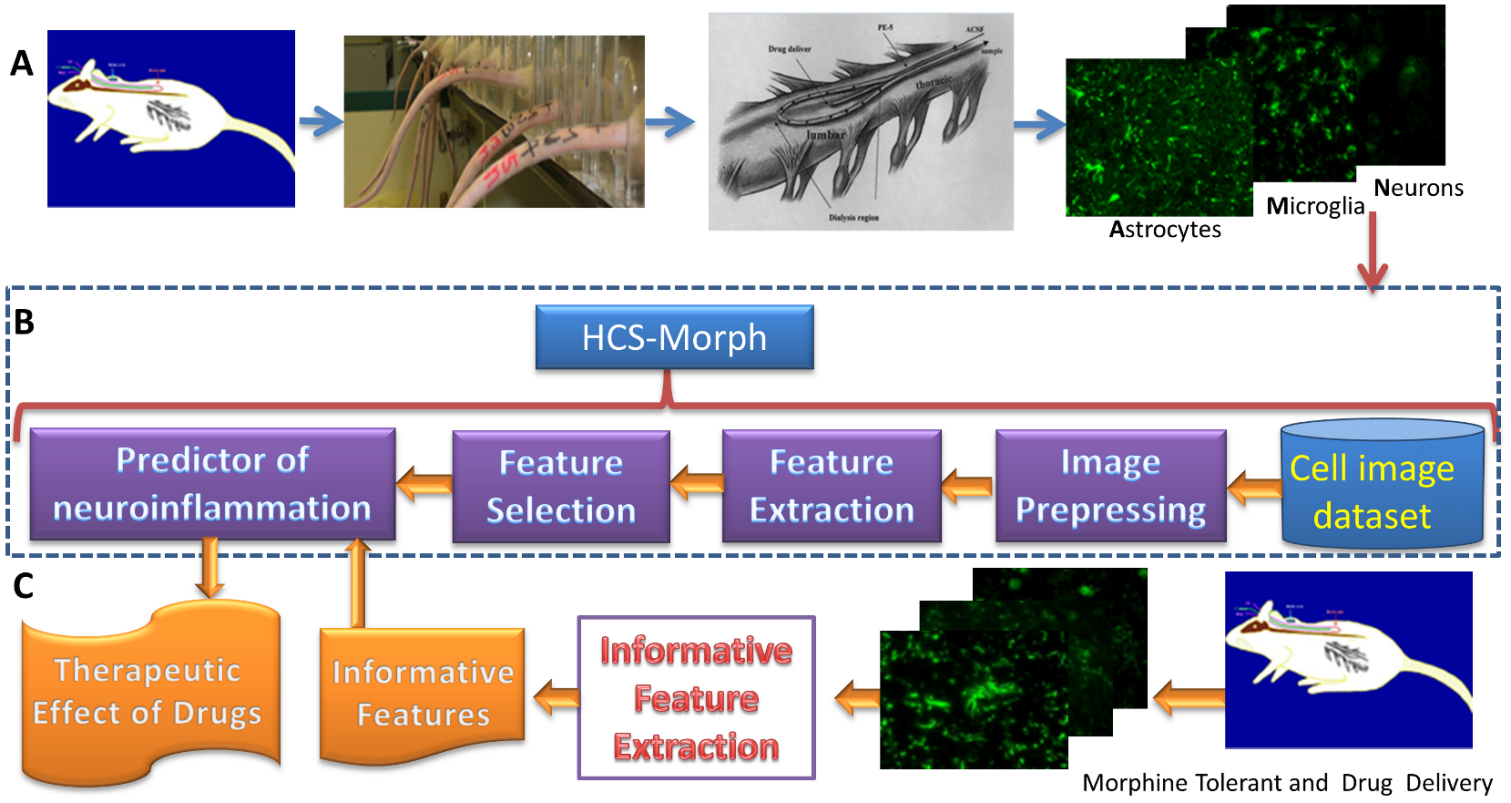
Flowchart of the proposed HCS-Morph method with materials. The computer-aided image diagnosis system facilitates developments of the tolerance therapy with anti-neuroinflammation drugs. A) Producing training images of neuroinflammation and non-neuroinflammation for astrocytes, microglia, and neurons. The procedure includes rat preparation and intrathecal drug delivery, anti-nociceptive test, spinal cord immunostaining sections, and image acquisition. B) Designing a prediction platform of neuroinflammation based on the HCS-Morph method. C) Evaluating effects of the tolerance therapy with anti-neuroinflammation drugs from immunostaining images.

The proposed experiments were approved by our animal use and care ethics committee in line with the principles of replacement, reduction and refinement. The use of rats conformed to the Guiding Principles in the Care and Use of Animals of the American Physiology Society and was approved by the National Defense Medical Center Animal Care and Use Committee of National Defense Medical Center in Taiwan. Fig 1 illustrates the proposed HCS-Morph method with materials.

#### Rat preparation and intrathecal drug delivery

While preparing animals for the implantation of the PE–10 intrathecal (i.t.) catheter (Becton Dickinson, Sparks, MD, USA), male Wistar rats (320–400 g) were anesthetized with phenobarbital (65 mg/kg, intraperitoneally; Sigma) and implanted with one i.t. catheter (8.5±0.5 cm) via the atlantooccipital membrane down to the lumbar enlargement (L1-L2) of the spinal bony structure. We used a polyethylene tube (8.5±0.5 cm in length, 0.008 in. ID, 0.014 in. OD) and a 3.5 cm silastic tube to construct intrathecal catheters. The polyethylene tube was inserted into the silastic tube and sealed with epoxy resin and silicon rubber. The end of the catheter was then externalized and fixed to the dorsal aspect of the head. The i.t. catheter was linked to a mini-osmotic pump and used to infuse morphine (15 μg/h), saline (1 μl/h), gabapentin (15 μg/h, Sigma), or morphine (15 μg/h) plus gabapentin (15 μg/h, Sigma) for five days. The morphine and saline groups were used for training the prediction model, and the gabapentin group is used for independent test of the model. After catheterization, all rats were returned to their home cages for rest. Each rat was housed individually and maintained on a 12 h light/dark cycle with food and water available. Rats with neurological deficits were excluded.

#### Anti-nociceptive test

The anti-nociceptive test is adjusted from previous studies [3,7]. One group of 15 rats received a constant morphine infusion for five days for tolerance development, while a control group of 15 rats received a constant saline infusion. An additional group of 10 rats received a co-infusion of morphine and gabapentin to evaluate the gabapentin effect on morphine tolerance development. All drug infusions were administered at a rate of 1μl/h through a mini-osmotic pump (Alzet, Cupertino, CA) implanted in the interscapular region. The development of morphine tolerance was assessed by a tail-flick latency test. Tail-flick latency using a hot water immersion test (52±0.5°C) was documented prior to drug infusion and once daily for 5 days after infusion began. The baseline tail-flick latency was 2±0.5 s and 10 seconds was allocated as the cutoff time to prevent tail injury. Rats were placed in plastic restraints during drug injection and the anti-nociception assay. The morphine analgesic effects were converted to a percentage of the maximal possible effect (%MPE) using the formula: MPE = [(test latency—baseline latency)/(maximum latency—baseline latency)]×100. We monitored the effect of gabapentin on morphine tolerance using the tail-flick latency test, and also used the immunohistochemistry method to detect the level of neuroinflammation related to the morphine and gabapentin co-infusion. An anti-nociception response curve was constructed for each study group.

The tail-flick latency test indicated the group receiving chronic intrathecal infusion of morphine developed morphine tolerance. The behavioral tail-flick assay on the 10 gabapentin-treated rats shows a significant attenuation of morphine tolerance (see the Results and Discussion section).

#### Spinal cord immunostaining sections and image acquisition

Following the completion of the experiments, all the rats were sacrificed by exsanguination under isoflurane anesthesia (ABBOTT, Abbott Laboratories Ltd., Queenborough, Kent, England). A laminectomy was performed at the lower level of the twelfth thoracic vertebra, and the L5–S3 segment of the spinal cord was removed and embedded in an optimal cutting temperature compound (Sakura Finetec Inc., USA) for immunohistochemistry.

Spinal cord sections (5μm) were fixed by soaking in ice-cold acetone/methanol (1:1) for 5 min. After three washes in ice-cold phosphate-buffered saline (PBS), sections were categorized by incubation overnight at 4°C with the FITC-labeled mouse monoclonal anti-glial fibrillary acidic protein (GFAP) antibody for astrocytes (Molecular Probes, Oregon, USA), the FITC-labeled mouse monoclonal anti-rat CD11b/c antibody for microglia (OX42; Serotec, Oxford, UK), and the FITC-labeled mouse monoclonal anti-neuronal nuclei for neurons (Chemicon, Temecula, CA) diluted in 1% normal goat serum in PBS. After three PBS rinses, 400x magnification images were taken using an Olympus BX 50 fluorescence microscope (Olympus Optical, Tokyo, Japan) and a Delta Vision disconsolation microscopic system operated using SPOT software (Diagnostic Instruments Inc., USA). For each rat, three images were produced for the three cells astrocytes, microglia, and neurons.

### Methods

The proposed HCS-Morph method uses an optimization approach to simultaneously identify informative features and create a prediction model for exploring neuroinflammation in morphine tolerance, and then establishes a computer-aided image diagnosis system to facilitate the development of tolerance therapy with anti-neuroinflammation drugs, as described below.

#### Image preprocessing

**Fig 2:**
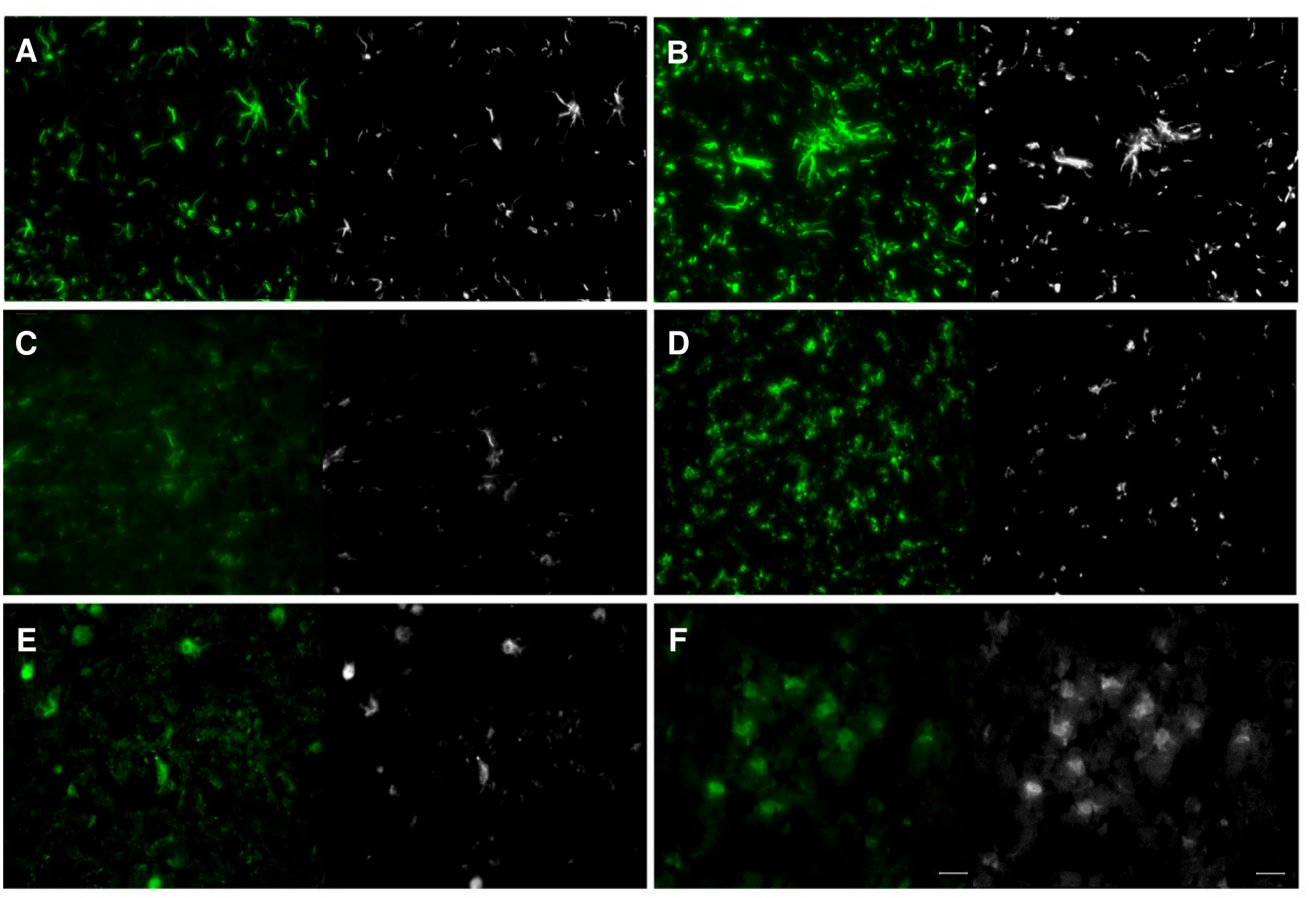
Typical images of control and morphine-tolerant cells in the spinal cord and their preprocessed images. (A) Astrocytes of the saline control. (B) Astrocytes with morphine tolerance. (C) Microglial of the saline control. (D) Microglial with morphine tolerance. (E) Neurons of the saline control. (F) Neurons with morphine tolerance. Scale bar represents 25μm.

The original immunostaining images are recorded in RGB format. The following preprocessing procedure was applied to the original images of cells in the spinal cord: 1) the color images are converted into gray scale and binary formats; 2) the scale of intensity is normalized by linear transformation into [0, 65,535]; 3) the image size is down-sampled from 1600×1200 to 800×600; 4) all objects smaller than 50 pixels are removed, and 5) background noise is removed by using the Otsu’s method [22]. Fig 2 shows example images of the saline controls and morphine-tolerant rats with neuroinflammation of cells in the spinal cord and their corresponding preprocessed images.

#### Image Feature extraction

Image-based high-content screening (HCS) has been shown to be a promising phenotypic screening approach for drug discovery. However, the majority of previous HCS-based studies (60–80%) made use of only one or two image-based features measured from each sample and disregarded the distribution of those features among each cell population [20]. Feature extraction and feature selection methods from HCS images play important roles in designing computer-aided image diagnosis systems [21]. To our best knowledge, no previous studies have reported effective feature identification methods for identifying the phenotypic change of neuroinflammation in the HCS images of cells in the spinal cord.

We comprehensively investigate numerous potential features that can be extracted from the readout of the HCS images to characterize phenotypic changes of cells with neuroinflammation activity. All the features can be categorized into five classes according to their properties (i.e., moment, texture, intensity, morphology, and frequency) which describe local or/and global properties of multiple cells in a highly multiplex immunostaining image. The design of the HCS-Morph method accounts for both the properties of individual cells and also the distribution properties of multiple cells in the whole image.

This work uses 21 feature sets belonging to the five classes as follows: 1) moment: geometric moments [23], Legendre moments [24], Tchebichef moments [25], Krawtchouk moments [26], Zernike moments [27], pseudo-Zernike moments [24], radial Tchebichef-Fourier moments [27], and Fourier-Mellin moments [28]; 2) texture: gray scaled co-occurrence matrix (GLCM) [29] and Haralick texture [29,30]; 3) intensity: histogram and relative histogram; 4) morphology: square model shape matrix [31], polar model shape matrix [32], and region properties [33], and 5) frequency: subcellular location feature SLF1 [34], generic Fourier descriptors [35], Granularity spectrum [36], Gabor filter [34], Wavelet energy (Daubechies4, Haar) [34], and Fourier transform [37]. The total number of candidate features in the 21 feature sets is *N* = 5,033, which can be roughly categorized into three classes as follows.

1. Neuronal features: Five features are commonly used in the literature, including Mean Cell Intensity, Cell Number, Total Cell Area, Largest Cell Area, and Largest Cell Diameter. Some neuronal morphology quantification methods can be used to extract the neuronal features [38,39]. These features are extractable, interpretable, and understandable in observing morphological changes of neuron cells [10,40,41].
2. Interpretable features: Interpretable features reveal their meanings directly from the whole images of cells such as intensity variation, morphology change, and texture homogeneity. They are directly computable and human-interpretable, e.g., the number of pixels in a specific range of intensity (“Pixel Area”) and the occurrence (“Texture Area”) and variation (“Texture Area Variation”) of pixel pairs that satisfy a specific condition.
3. Computational features: These features describe global image properties with complicated mathematic functions such as Polar-coordinate-based moments, Cartesian-coordinate-based moments, Haralick texture, Fourier descriptors, and wavelet energy. The features are not easily observed directly but may effectively respond to phenotypic changes of cells in the spinal cord.

#### Image feature selection

In developing an effective method for identifying HCS-based features to predict neuroinflammation in morphine tolerance, this work considers the following facts. First, because no prior knowledge is utilized, many potential features (*N* = 5,033) in the HCS images are extracted for comprehensive investigation. Second, simultaneous selection of multiple features generally provides better prediction accuracy than the combination of features individually selected by univariate analysis [42]. Third, biologists and clinical researchers require not only high prediction accuracy but also high feature interpretability. This work proposes a coarse-to-fine feature selection approach to obtain a small set of informative and easily-interpretable features for predicting neuroinflammation in morphine tolerance. The proposed neuroinflammation feature selection method consists of the two steps: univariate feature selection and multivariate feature selection, as described below. The same feature selection method is applied to each type of the three cells in the spinal cord.

1. Univariate feature selection. The p-value (*P*) for each of *N* candidate features is calculated using *t*-test analysis to identify the significant features for distinguishing neuroinflammatory and non-neuroinflammatory cells. The feature set of size *n* obtained from *N* features in this coarse step consists of three classes: 1) the best *n*_1_ features according to p-value, 2) the best *n*_2_ features considering both p-value and human interpretability, and 3) *n*_3_ neuronal features reported in the literature to characterize the cells in the spinal cord. In this work, *n*_1_ = 15, *n*_2_ = 20, *n*_3_ = 5, and *n* = 40. The *n*_1_ features may come from the sets of neuronal, interpretable, and computational features. The *n*_2_ interpretable features are selected with human intervention. The above-mentioned five neuronal features of morphology (*n*_3_ = 5) are adopted.
2. Multivariate feature selection. Multivariate feature selection identifies a small set of *m* features responding to the phenotypic change by maximizing prediction accuracy for neuroinflammation. An intelligent bi-objective combinatorial genetic algorithm (IBCGA) is used to solve the combinatorial optimization problem of C(*n*, *m*) having a huge search space of size C(*n*, *m*) = *n*!/(*m*!(*n*-*m*)!). The IBCGA uses an intelligent genetic algorithm (IGA) [43] with an inheritance mechanism [42] to efficiently search for the solution *S*_r+1_ to C(*n*, *r*+1) by inheriting the good solution *S*_r_ to C(*n*, *r*). The IGA algorithm based on orthogonal experimental design uses a divide-and-conquer strategy and a systematic reasoning method instead of the conventional generate-and-test method to efficiently solve the large-scale combinatorial optimization problem. The IBCGA algorithm solves the combinatorial optimization problem by minimizing the number of selected features and maximizing the fitness function *f*(*S*) defined as the prediction accuracy of 10-fold cross-validation (10-CV) for the candidate solution *S*. The design of the prediction model is to simultaneously determine the optimal parameter settings of the support vector machine (SVM) and feature selection. The input for the SVM-based model design procedure is a training dataset of two-class images (neuroinflammation and non-neuroinflammation) and the output contains a set of *m* selected image features and an SVM classifier with associated parameter settings of *γ* and *C*. A radial basis kernel function exp(-*γ*||*x*_i_—*x*_j_||^2^) is adopted, where *x*_i_ and *x*_j_ are training samples, and *γ* is a kernel parameter [44]. In this work, *γ* ∈{2^−7^, 2^−6^, …, 2^8^} and *C*∈{2^−7^, 2^−6^, …, 2^8^}. The input and output of the used IBCGA algorithm are the training samples of *n* features and *m* selected features, respectively.

The IBCGA with the fitness function *f*(*S*) can simultaneously obtain a set of solutions, *S*_*r*_, where *r* = *r*_start_, *r*_start_+1, …, *r*_end_ in a single run. In this work, the parameter settings are *r*_start_ = 2, *r*_end_ = 30, *N*_pop_ = 60, *p*_*c*_ = 0.8, *p*_*m*_ = 0.05, and *Gmax* = 60. The customized IBCGA algorithm for the image feature selection is given below.

Step 1: (Initiation) Randomly generate an initial population of *N*_pop_ individuals. All the *n* binary genes *f*_i_ have *r* 1’s and *n*-*r* 0’s where *r* = *r*_start_.
Step 2: (Evaluation) Evaluate the fitness values of all individuals using *f*(*S*).
Step 3: (Selection) Use a conventional tournament selection that selects the winner from two randomly selected individuals to form a mating pool.
Step 4: (Crossover) Select *p*_*c*_·*N*_pop_ parents from the mating pool to perform orthogonal array crossover [43] on the selected pairs of parents where *p*_*c*_ is the crossover probability.
Step 5: (Mutation) Apply a conventional mutation operator to the randomly selected *p*_*m*_·*N*_pop_ individuals (except the best individual) in the new population where *p*_*m*_ is the mutation probability.
Step 6: (Termination test) If the stopping condition of performing *Gmax* generations for obtaining the solutions *S*_*r*_ is satisfied, output the best individual as *S*_*r*_. Otherwise, go to Step 2.
Step 7: (Inheritance) If r < *r*_end_, randomly change one bit in the binary genes *f*_i_ for each individual from 0 to 1; increase the number *r* by one, and go to Step 2.
Step 8: (robustness) Perform Steps 1–7 for *R* (= 30 in this work) independent runs and obtain the best one of *R* solutions.

The best solution can be determined by considering the most accurate one *S*_a_ with the highest fitness value or the robust one *S*_r_ with the highest appearance score [45]. The appearance score considers both the fitness value and the mean number of times for individual features selected in the *R* runs. In this work, the solution *S*_r_ was adopted.

#### Prediction platform for neuroinflammation

Neuroinflammation activity plays an important role in the development of morphine tolerance [5,46]. The role of neuroinflammation in the development of morphine tolerance and the effect of the TNF-α inhibitor, etanercept, in the attenuation of morphine tolerance are explained in the review article [2]. The proposed HCS-Morph method uses an SVM-based classifier with informative image features to predict images of astrocytes, microglia, and neurons in the spinal cord. Informative image features should have good prediction ability to respond to phenotypic changes caused by neuroinflammation. Interpretability of the informative features should be also considered to gain an insight into phenotypic changes of cells in the spinal cord. Moreover, interpretable features relating to neuroinflammation can moderately cope with the problem of high-level background noise. A related study of the identification of phenotypic changes in multi-neuron images upon drug treatments with high-content screening can be found in our previous work [21]. The present work aims to identify sets of HCS-based neuronal features to establish an accurate prediction platform for neuroinflammation by solving the bi-objective optimization problem. To make the best use of all available samples, 15 controls and 15 morphine-tolerant rats were used to train the HCS-Morph model for establishing the neuroinflammation prediction platform.

Since neuroinflammation prediction has relatively lower cost (in terms of rats, experimental material, time, etc.) than traditional experimental approaches, the platform can benefit drug screening processes through minimizing the need for drug-treated rats. Once potential drugs are discovered, conventional experiments can be used for further validation of the drugs. Therefore, the computer-aided image diagnosis system based on HCS-Morph with accurate prediction of neuroinflammation can greatly facilitate the development of tolerance therapy with anti-neuroinflammatory drugs.

## Results

### Effects of intrathecal co-infusion of morphine and gabapentin

**Fig 3:**
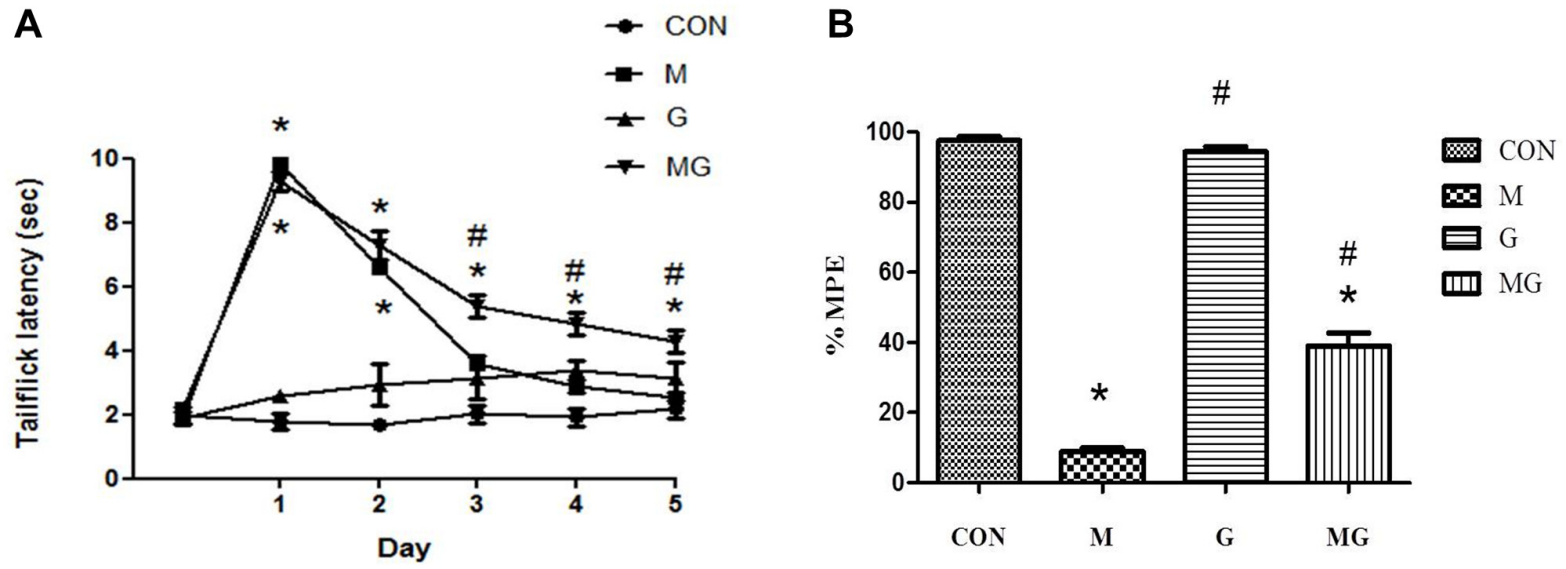
Anti-nociceptive effects of intrathecal co-infusion of morphine and gabapentin in chronic morphine-infused rats. The denotation * (*P*<0.05) is the comparison to the control group and # (*P*<0.05) is the comparison to the morphine-infused group. (A) Time course of tail-flick latencies over the 5-day period of i.t. infusion of saline or morphine with/without gabapentin. CON: saline (1 μl/h) infusion (n = 10); M: morphine (15 μg/h) infusion (n = 10); G: gabapentin (15 μg/h) infusion (n = 10); MG: morphine (15 μg/h) plus gabapentin (15 μg/h) co-infusion (n = 10). All data points are expressed as mean ± SEM. Co-infusion of morphine and gabapentin attenuates morphine tolerance formation. (B) Anti-nociceptive effect of a single challenge dose of morphine administrated in the rats on day 5. A single dose of morphine (15 μg) was administrated at 4 h after discontinuing drug infusion via mini-osmotic pump (when the basal tail-flick latencies had declined below 3s before morphine challenge in all test rats). The anti-nociceptive effect in the hot-water immersion (52°C) test was measured every 30 min for 180 min after morphine injection (15 μg) (n = 5 in each group). The maximal anti-nociceptive effect of morphine (15 μg) challenge in each group is expressed as the % change in the MPE compared to the saline control group (100% MPE).

Fig 3 shows the anti-nociceptive effects of intrathecal co-infusion of morphine and gabapentin on chronic morphine-infused rats. The number of animals used in each of the four groups is 10 and all the animals were injected by intrathecal infusion pump. Fig 3A reveals the attenuation of morphine tolerance formation using this co-infusion regiment. The intrathecal administration of gabapentin (15 μg/h) alone (G) did not produce an anti-nociceptive effect throughout the 5-day infusion as measured by the 52°C hot water immersion test. In morphine-infused rats (M), the maximal anti-nociceptive effect of morphine was detected on day 1 after beginning the intrathecal morphine infusion, while morphine tolerance was detected on day 2 and reached its maximum level on day 3. In contrast, the anti-nociceptive effects of the intrathecal morphine alone (M) and the co-infusion (MG) were significantly different from day 3. The anti-nociceptive effect of morphine was partially preserved when gabapentin (15 μg/h) was co-infused during tolerance induction.

As shown in Fig 3B for the anti-nociceptive effects on day 5, the morphine challenge (15 μg/10 μl, i.t.) at 4 h after cessation of drug infusion produced a significant anti-nociceptive effect in both the saline (CON) and gabapentin alone (G) infusion groups, but not in the morphine-infused group (M); however, co-infusion of gabapentin (MG) prevented a decline in the anti-nociceptive effect of morphine. The value of MPE was 97.5 ± 1.1 in saline-treated rats, 8.9 ± 0.9 in morphine-tolerant rats, 94.3 ± 1.5 in gabapentin-infused rats, and 39.0 ± 3.6 in the co-infused group. These results suggest that the gabapentin co-infusion with morphine significantly inhibits the development of morphine tolerance. This attenuation of morphine tolerance results from the prevention of neuroinflammation by the co-infusion of gabapentin and morphine.

Gabapentin is an effective drug used for the treatment of neuropathic pain with antihyperalgesic properties. In this study, intrathecal administration of gabapentin (15 μg/h) alone (G) did not produce an anti-nociceptive effect by tail flick test. It is consistent with the result found by Kilic et al. [47] that mice received gabapentin 10 mg/kg or 100 mg/kg intraperitoneally did not show an anti-nociceptive effect by tail flick test. Moreover, gabapentin could show its anti-nociceptive effect in 30 mg/kg intraperitoneally in neuropathic pain condition. It strongly indicated the effect of gabapentin should be to stabilize the nerve activity when nerve in neuropathy condition such as inflammation or post injury.

### Univariate feature extraction

To design an accurate HCS-Morph predictor with informative features for the three types of cells, we first filtered 40 candidate features out of 5,033 features obtained from 21 image feature descriptors using univariate feature selection. Three sets of the 40 candidate features, including their descriptions, are given in S1–S3 Tables for astrocytes, microglia, and neurons, respectively.

Between the two groups of controls and morphine-tolerant rats, 34 of the 40 candidate features of astrocytes are significantly different (*P* < 0.001), except for the two neuronal features, Cell Number (*P* = 0.08) and Largest Cell Area (*P* = 0.02), and four interpretable features (*P* < 0.01). Of the 40 features of microglia, 35 have *P* < 0.01 excluding the five neuronal features. The five neuronal features of neurons have *P* > 0.05 suggesting that these features do not provide sufficient information to predict neuroinflammation.

To further analyze the effectiveness of the 40 individual features on each of the three types of cells, the top–5 features with the smallest *P* values are listed in Tables 1, 2 and 3, respectively, for astrocytes, microglia, and neurons. In the Jackknife test, each image is used in rotation as an independent test while the other 29 images were used as a training set to design SVM-based predictors. The prediction result of the 30 tests is the Jackknife test accuracy. The Jackknife test was performed on the SVM-based predictors using a single feature to test the 30 rats belonging to the two classes, i.e., neuroinflammation and non-neuroinflammation.

**Table 1:** The top–5 features with the smallest *P* values for astrocytes.

Feature Name	Image Type Binary/Gray	Feature type	p-value (*P*)	Jackknife test (%)
1. Cell Distribution X/Y (0,2)	B	Interpretable	2.09E-09	96.67
2. Texture Information (1)	G	Interpretable	1.97E-08	86.67
3. Pixel Area (11)	B	Interpretable	2.20E-08	86.67
4. Radial Tchebichef Fourier (Binary, 0,0)	B	Computational	2.32E-08	96.67
5. Pixel Area Ring (121)	B	Interpretable	2.45E-08	96.67
Mean accuracy				92.67

**Table 2:** The top–5 features with the smallest *P* values for microglia.

Feature Name	Image Type Binary/Gray	Feature type	p-value (*P*)	Jackknife test (%)
1. Legendre (Gray, 0,2)	G	Computational	2.08E-05	83.33
2. Tchebichef (Gray, 0,4)	G	Computational	2.15E-05	83.33
3. Texture Area Variation (5,5,4)	G	Interpretable	4.80E-05	76.67
4. Texture Area Variation (3,5,4)	G	Interpretable	5.86E-05	80.00
5. Legendre (Binary, 0,4)	B	Computational	1.57E-04	80.00
Mean accuracy				80.67

**Table 3:** The top–5 features with the smallest *P* values for neurons.

Feature Name	Image Type Binary/Gray	Feature type	p-value (*P*)	Jackknife test (%)
1. Radial Tchebichef-Fourier (Gray, 0,15)	G	Computational	5.95E-04	70.00
2. Legendre (Gray, 1,15)	G	Computational	6.19E-04	76.67
3. Tchebichef (Gray, 1,15)	G	Computational	6.41E-04	76.67
4. Pseudo Zernike (Binary, 16,1)	B	Computational	7.24E-04	66.67
5. Legendre (Binary, 1,15)	B	Computational	7.29E-04	73.33
Mean accuracy				72.67

**Fig 4:**
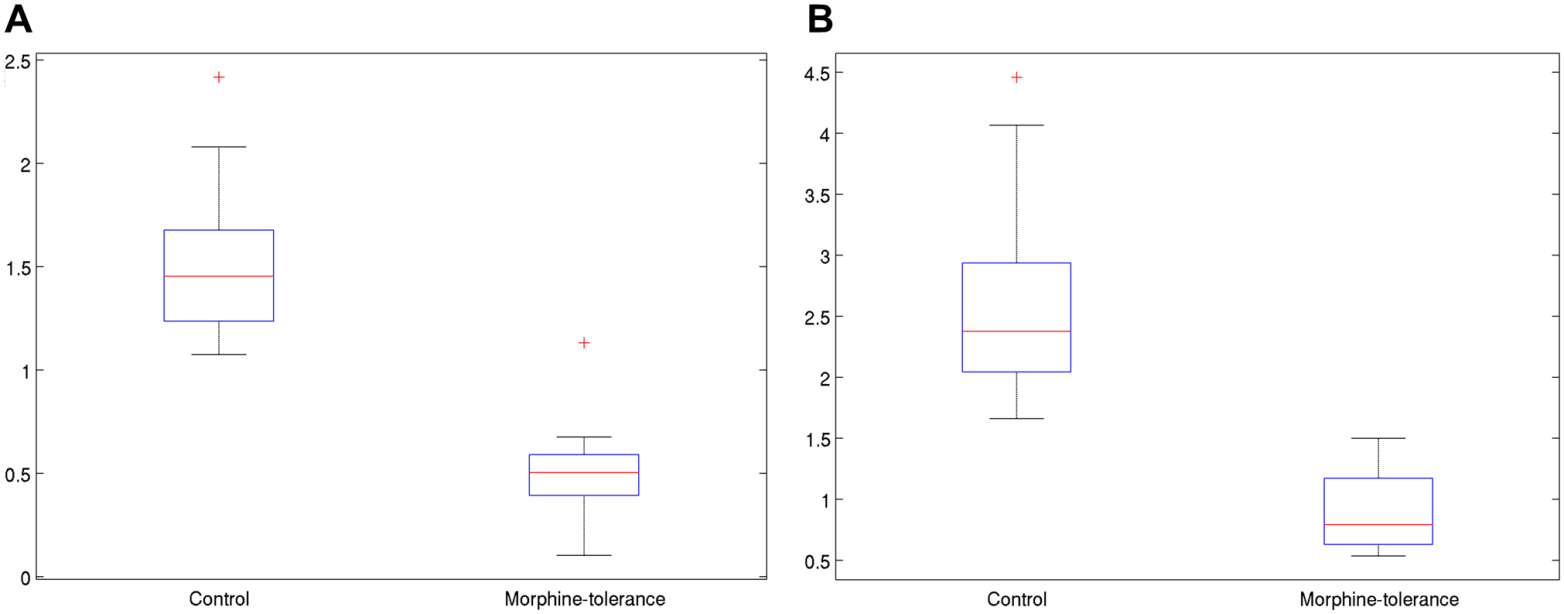
The statistics of M_02_ (left) and M_20_ (right) for the control and morphine-tolerance groups of astrocytes. The values of the morphine-tolerance group were significantly smaller than those of the control group for both M_02_ and M_20_.

As shown in Table 1, of the five features, four are interpretable and one is computational. Four features were extracted from binary images, and one from gray images. All five features have high levels of prediction accuracy (i.e., greater than 86.67% with a mean accuracy of 92.67%). The interpretable feature Cell Distribution X/Y (0, 2) with *P* = 2.09E-09 has the best prediction accuracy of 96.67% for astrocytes. The Cell Distribution X/Y (*i*, *j*) feature is one type of image moments, called a geometric moment, computed from a weighted mean of the pixel intensity [23]. The equation for calculating the geometric moments M_ij_ is as follows:

$$M_{ij}=\sum_{x=1}^{Nx}\sum_{y=1}^{Ny}x^{i}y^{j}I(x,y)$$ (1)

where *I*(x, y) is the intensity of a pixel at the coordinate (x, y). For binary images, the intensity equals 0 or 1. The variables *i* and *j* are the moment orders. In this work, *Nx* = 800 and *Ny* = 600. In calculating the feature Cell Distribution X/Y (0, 2) (i.e., M_02_), *i* = 0 and *j* = 2. The center (M_10_/M_00_, M_01_/M_00_) of the fluorescence area was translated to the center of the image for translation normalization. The feature value was divided by a suitable scale factor relating to the total number of fluorescence pixels for scale normalization. This feature is highly correlated to the distribution of fluorescent pixels. Remarkably, the averaged Pearson’s correlation coefficient between M_02_ and M_20_ reaches 0.854. Considering the rotation invariants, the features M_02_ and M_20_ represent the same distribution of fluorescent pixels if the vertical and horizontal axes are exchanged. Fig 4 show the statistics of M_02_ and M_20_ for the control and morphine-tolerance groups of astrocytes. The values of the morphine-tolerance group were significantly smaller than those of the control group for both M_02_ and M_20_. The feature Cell Number has a *P* value of 0.080, suggesting that the cell numbers between control and morphine-tolerance groups were not significantly different. The decreased moments reveal that astrocytes in neuroinflammation would be aggregated compared with astrocytes in the control group with no neuroinflammation.

In Table 2, the best feature for microglia is one of Legendre moments [24], Legendre (Gray, 0, 2), with *P* = 2.08E-05 and accuracy of 83.33%. There are two interpretable features and three computational features. All five features have prediction accuracies greater than 76.67% with a mean accuracy of 80.67%. For the five features of neurons shown in Table 3, the best feature is the computational feature Radial Tchebichef Fourier (Gray, 0, 15) with *P* = 5.05E-04 and accuracy of 70.00%. The five features are all computational features with a mean accuracy of 72.67%. The p-values of the feature M_02_ for microglia and neurons are *P* = 0.0811 and 0.0615, respectively, suggesting that the second-order geometric moments are not significantly different. The results reveal that neuroinflammatory microglia (neurons) would not lead to significant aggregation, compared with non-neuroinflammatory microglia (neurons).

Table 4 shows the performance of each neuronal feature for the prediction of neuroinflammation in the images of astrocytes, microglia, and neurons in terms of *P* value and prediction accuracy. The results show that the three neuronal features, Mean Cell Intensity, Total Cell Area, and Largest Cell Diameter have the smallest p-values (P<0.001) for astrocytes, but not for both microglia and neurons. The two features Mean Cell Intensity and Total Cell Area have an accuracy of 90.00% for astrocytes. The feature Cell Number is not significantly different (P>0.05) between images of neuroinflammation and non-neuroinflammation for the three types of cells. The increase of total cell area with no significant change of cell number reveals the cell extension of astrocytes when neuroinflammation occurs. Notably, the feature Mean Cell Intensity has *P* = 0.024 (<0.05) for microglia. The feature Mean Cell Intensity responses the neuroinflammation activity of cells in the immunostaining images. The results suggest that neuroinflammation activity expresses on phenotypic changes more predominantly in the order: astrocytes, microglia and neurons. The feature Total Cell Area has *P* = 0.563 (>0.05) for microglia. Therefore, using the two neuronal features Mean Cell Intensity and Total Cell Area, morphological changes due to neuroinflammation are more easily detected on astrocytes than on both microglia and neurons.

**Table 4:** The *P* values and prediction accuracies (%) of the neuronal features for astrocytes, microglia, and neurons.

Feature Name	Astrocytes	Microglia	Neurons
1. Mean Cell Intensity	2.08E-06 (90.00)	0.024 (60.00)	0.350 (56.67)
2. Cell Number	0.080 (63.33)	0.290 (30.00)	0.104 (40.00)
3. Total Cell Area	6.64E-07 (90.00)	0.563 (46.67)	0.054 (60.00)
4. Largest Cell Area	0.020 (40.00)	0.078 (36.67)	0.561 (70.00)
5. Largest Cell Diameter	2.19E-05 (66.67)	0.538 (46.67)	0.328 (70.00)

### Multivariate feature selection and prediction accuracy

We used a multivariate feature selection method to identify a minimal set of informative features from the 40 candidates feature for each cell type to design SVM-based predictors for predicting neuroinflammation from immunostaining images of astrocytes, microglia, and neurons. Table 5 shows performance comparisons between two sets of machine-readable and human-readable features for the three cell types. The Jackknife test accuracies of the SVM-Neuron method using the sets of five neuronal features are 86.67%, 43.33% and 56.67% for astrocytes, microglia, and neurons, respectively. The prediction accuracies of HCS-Morph using the feature set selected by IBCGA for astrocytes, microglia, and neurons are 96.67%, 90.00%, and 86.67%, respectively, with mean feature numbers 1.0, 3.0, and 4.0. The typical feature sets of HCS-Morph considering high accuracy and robustness are shown in Table 6, as described below. The prediction of astrocytes uses a single feature Cell Distribution X/Y (0, 2) to yield an accuracy of 96.67%. The prediction of microglia uses three features, Krawtchouk (Binary, 11, 16), Texture Area Variation (3, 5, 4), and Legendre (Binary, 2, 4) to yield an accuracy of 90.00%. Krawtchouk (Binary, 11, 16) is a computational feature with the highest selection frequency in 30 independent runs using Cartesian-coordinate-based moments relating to cell distribution. The prediction of neurons uses one interpretable feature Different Pixel Area (30) (i.e., the number of pixels in a specific range of intensity) and three computational features, Tchebichef (Gray, 10, 12), Legendre (Binary, 15, 5), and Legendre (Gray, 15, 5) to yield an accuracy of 86.67%. Considering both machine-readable and human-readable features, astrocytes are the best target for choice to predict neuroinflammation in morphine tolerance.

**Table 5:** Jackknife tests (%) and the mean feature numbers of the SVM-based predictors for 30 samples.

Method	Astrocytes (feature number)	Microglia (feature number)	Neurons (feature number)
SVM-Neuron	86.67 (5)	43.33 (5)	56.67 (5)
HCS-Morph	96.67 (1.0)	90.00 (3.0)	86.67 (4.0)

**Table 6:** Typical feature sets using the feature selection algorithm IBCGA from 30 training samples.

Cell types	Feature name	Feature type
1. Astrocytes	1. Cell Distribution X/Y (2, 0)	Interpretable
2. Microglia	1. Krawtchouk (Binary, 11, 16)	Computational
	2. Texture Area Variation (3, 5, 4)	Interpretable
	3. Legendre (Binary, 2, 4)	Computational
3. Neurons	1. Different Pixel Area (30)	Interpretable
	2. Tchebichef (Gray, 10, 12)	Computational
	3. Legendre (Binary, 15, 5)	Computational
	4. Legendre (Gray, 15, 5)	Computational

The HCS-Morph method investigates comprehensive features to predict neuroinflammation in morphine tolerance for astrocytes, microglia, and neurons, including the features related to cell size, cell density, cell distribution, cell texture, and cell intensity of immunostaining images. The above-mentioned experimental results derive the following summary. The set of the three features, Mean Cell Intensity, Total Cell Area, and Cell Distribution X/Y (0, 2), respectively relevant to cell communication, cell extension, and cell migration, is promising for designing the proposed computer-aided image diagnosis system. To predict neuroinflammation for microglia and neurons, the sets of machine-readable features in Table 6 are suitable to design the image diagnosis system.

### Therapeutic effect of gabapentin and platform of drug discovery

The experiments shown in Fig 4 suggest that the gabapentin co-infusion with morphine inhibits the development of neuroinflammation and thus attenuates morphine tolerance formation. Therefore, immunostaining images of the co-infused cells should be predicted as a non-neuroinflammation class. The prediction accuracies with the numbers of features used on the 10 co-infused rats are shown in Table 7. The prediction accuracy of 100% for astrocytes suggests that the used three features are indeed a good feature set for predicting neuroinflammation and astrocytes is the best choice to assess the neuroinflammation activity. The prediction accuracies of microglia and neurons were 90% and 60%, respectively, suggest that the images of microglia are more suitable for predicting neuroinflammation than those of neurons. By additionally considering the mean cell intensity relating to neuroinflammation activity, the results suggest that neuroinflammation activity on phenotypic changes expresses more predominantly in astrocytes and microglia than in neurons. Proinflammatory cytokines act on astrocytes and microglia to respond the stimuli with morphological changes more than on neurons from the computer-aided image diagnosis system based on the prediction of neuroinflammation.

**Table 7:** Prediction accuracies on the 10 gabapentin co-infused rats.

	Astrocytes	Microglia	Neurons
Test accuracies (%)	100	90	60
Feature number	3	3	4

The analysis of immunoreactivity by HCS-Morph with identified informative features indeed facilitates the recognition of morphological changes of the three cell types and the effect of treated drugs on neuroinflammation. Since neuroinflammation would attenuate morphine’s analgesic effects, drugs that inhibit neuroinflammation could have potential for use in treating neurodegeneration disease. The investigation of the therapeutic effect provides the first evidence for the role of gabapentin in the attenuation of morphine tolerance from phenotypic changes of astrocytes and microglia.

For high neuroinflammation activity consistent with the decay of the opioid analgesic effect, we might carefully apply this image diagnosis platform to screen for the therapeutic potential of other drugs to improve opioid analgesia through neuroinflammation activity. The proposed platform provides several advantages such as minimizing rate usage, time savings, and fast evaluation of neuronal activity in the laboratory. Moreover, this platform could facilitate the investigation of therapeutic effects and screening of potential drugs with an anti-inflammatory effect for treatment of multiple sclerosis, Parkinsonism disorder and other degenerative neuronal diseases accompanied by significant neuroinflammation.

## Discussion

This work investigates HCS-based features for quantifying phenotypic changes responding to neuroinflammation in morphine tolerance and develops the first practical, low-cost computer-aided image diagnosis system to greatly facilitate the development of tolerance therapy with anti-neuroinflammation drugs. Phenotypic changes may include increases or decreases in the production of cellular products such as proteins and/or changes in the morphology of the cells in the spinal cord. The resolution of used immunostaining images is not high enough to extract detailed features of a cell such as soma size, neurite length, and neurite number. However, the used systematic approach to feature selection can identify a set of interpretable and neuronal features responding to neuroinflammation in morphine tolerance. A concise explanation of the implications of the findings consistent with related studies is given below for gaining an insight into neuroinflammation in morphine tolerance.

Long-term morphine treatment may lead to neuroinflammation with morphine tolerance. Chronic neuroinflammation is the continual activation of glial cells including astrocytes and microglia. Once activated, astrocytes may release various growth factors, increase synthesis of GFAP, and undergo morphological changes to form a glial scar [48]. GFAP plays an important role in astrocyte-neuron interactions as well as cell-cell communication. Reactive astrocytes form a dense web of their plasma membrane extensions that fills the empty space generated by the dead or dying neuronal cells. The experimental results gained an insight into the reaction of astrocytes in response to neuroinflammation in morphine tolerance by observing the following three evidences, which are consistent with related studies. 1) The mean cell intensity was enhanced in the immunostaining images of astrocytes using the anti-GFAP antibody suggesting that mechanical strength of astrocytes was enhanced for astrocyte-neuron communication. Zhang *et al*. found that neurons could actively release transmitters to produce bidirectional communication between neurons and surrounding satellite glial cells [49]. 2) The total cell size and largest cell diameter were increased suggesting that reactive astrocytes underwent morphological changes to form a glial scar to reestablish the physical and chemical integrity of the spinal cord. Glial scarring following inflammation after spinal cord injury is due to an extreme, uncontrolled form of reactive astrogliosis that typically occurs around the injury site [48]. 3) The reduced second-order geometric moment suggests that neuroinflammation of the spinal cord induced the migration of astrocytes to sites of spinal cord lesions. Li et al. has proposed the activation of the ERK1/2 signaling pathway to mediate astrocyte migration and glial scar formation in response to spinal cord injury [50].

Microglia are the resident macrophages that act as the first and main form of active immune defense in the central nervous system [51]. In the case of long-term morphine treatment, microglia reacts rapidly to decrease neuroinflammation to prevent potentially fatal damage of the spinal cord. The CD11b antigen is expressed on microglia. From the informative features, some observations are consistent with related studies discussed below. The mean cell intensity was enhanced (P = 0.024) in the immunostaining images of microglia using the anti-rat CD11b/c antibody suggesting that microglia were significantly activated. Microglia are the second most prominent cell type that exist within the glial scar [48]. Microglia are rapidly activated via various proinflammatory cytokines near the sites of spinal cord lesions. When microglia finds any foreign material, they move through their set region to phagocytose the material. Unlike the obvious morphological changes of reactive astrocytes, the total cell area (P = 0.563) and the largest cell diameter (P = 0.538) of activated microglia were not significantly changed. Instead, the most effective features are the class of moments such as geometric moments, Legendre moments, Tchebichef moments, and Krawtchouk moments that are relevant to cell distribution.

Chronic inflammation may cause the degradation of tissue. Astrocytes become activated in response to signals released by injured neurons or activated microglia. Neurons release neurotransmitters to produce bidirectional communication with surrounding glial cells [49]. The morphological change of neurons was not obvious. All the neuronal features (P>0.05) in the immunostaining images of neurons were not informative enough to predict neuroinflammation. The experimental results suggest that neuroinflammation activity expresses predominantly in astrocytes and microglia than in neurons, which is consistent with previous findings [5,9,52].

## Conclusions

Chronic morphine treatment produces progressive tolerance which attenuates analgesic effect and impedes clinical utilization. Neuroinflammation activity plays an important role in the development of morphine tolerance. This work proposes an automation method HCS-Morph to analyze immunostaining images of cells in the rat spinal cord for: 1) identifying and analyzing informative HCS features upon the response to phenotypic changes given morphine tolerance with neuroinflammation for astrocytes, microglia, and neurons; 2) establishing a mathematic model for neuroinflammation prediction to assess the effect of gabapentin on morphine tolerance therapy, and 3) developing a computer-aided image diagnosis system based on the neuroinflammation prediction to greatly facilitate the development of tolerance therapy with anti-neuroinflammatory drugs (such as gabapentin).

The informative features to predict neuroinflammation in morphine tolerance for astrocytes, microglia, and neurons are relevant to cell size, cell density, cell distribution, cell texture, and cell intensity. Experimental results suggest that neuroinflammation activity expresses more predominantly in astrocytes and microglia than in neuron cells. The most effective set of features for predicting neuroinflammation of astrocytes comprises mean cell intensity, total cell area, and second-order geometric moment, relevant to cell communication, cell extension, and cell migration, respectively. Moreover, the investigation of the therapeutic effect provides the first evidence for the role of gabapentin in the attenuation of morphine tolerance from phenotypic changes of astrocytes and microglia. The expression levels of neuroinflammation in rat spinal cords predicted by the HCS-based platform are relevant to anti-nociceptive behavior in rats. Thus, the proposed platform can be used to not only monitor the pharmacological effect in the central nerve system but also change the neuroinflammation profile. The platform could potentially be applied to other neuroinflammation animal models related to neuronal degeneration diseases such as Amyotrophic lateral sclerosis, Parkinson's disease, multiple sclerosis, and Alzheimer's disease.

## Supporting Information

S1 Table The 40 features of univariate feature selection for astrocytes. (DOCX)

S2 Table The 40 features of univariate feature selection for microglia. (DOCX)

S3 Table The 40 features of univariate feature selection for neurons. (DOCX)

## References

[1] Koob GF, Bloom FE (1988) Cellular and molecular mechanisms of drug dependence. Science 242: 715–723. 2903550 10.1126/science.2903550

[2] Shen CH, Tsai RY, Wong CS (2012) Role of neuroinflammation in morphine tolerance: effect of tumor necrosis factor-alpha. Acta Anaesthesiol Taiwan 50: 178–182. 23385041 10.1016/j.aat.2012.12.004

[3] Trujillo KA, Akil H (1991) Inhibition of morphine tolerance and dependence by the NMDA receptor antagonist MK–801. Science 251: 85–87. 1824728 10.1126/science.1824728

[4] Tai YH, Wang YH, Tsai RY, Wang JJ, Tao PL, Liu TM, et al (2007) Amitriptyline preserves morphine's antinociceptive effect by regulating the glutamate transporter GLAST and GLT–1 trafficking and excitatory amino acids concentration in morphine-tolerant rats. Pain 129: 343–354. 17346885 10.1016/j.pain.2007.01.031

[5] Song P, Zhao ZQ (2001) The involvement of glial cells in the development of morphine tolerance. Neurosci Res 39: 281–286. 11248367 10.1016/s0168-0102(00)00226-1

[6] Merighi S, Gessi S, Varani K, Fazzi D, Stefanelli A, Borea PA (2013) Morphine mediates a proinflammatory phenotype via mu-opioid receptor-PKCvarepsilon-Akt-ERK1/2 signaling pathway in activated microglial cells. Biochem Pharmacol 86: 487–496. 10.1016/j.bcp.2013.05.027 23796752

[7] Lin SL, Tsai RY, Tai YH, Cherng CH, Wu CT, Yeh CC, et al (2010) Ultra-low dose naloxone upregulates interleukin–10 expression and suppresses neuroinflammation in morphine-tolerant rat spinal cords. Behav Brain Res 207: 30–36. 10.1016/j.bbr.2009.09.034 19799935

[8] Ikeda H, Miyatake M, Koshikawa N, Ochiai K, Yamada K, Kiss A, et al (2010) Morphine modulation of thrombospondin levels in astrocytes and its implications for neurite outgrowth and synapse formation. Journal of biological chemistry 285: 38415–38427. 10.1074/jbc.M110.109827 20889977 PMC2992274

[9] Harada S, Nakamoto K, Tokuyama S (2013) The involvement of midbrain astrocyte in the development of morphine tolerance. Life sciences 93: 573–578. 10.1016/j.lfs.2013.08.009 23988850

[10] Mattioli TA, Milne B, Cahill CM (2010) Ultra-low dose naltrexone attenuates chronic morphine-induced gliosis in rats. Mol Pain 6: 22 10.1186/1744-8069-6-22 20398374 PMC2862024

[11] Stone LS, German JP, Kitto KF, Fairbanks CA, Wilcox GL (2014) Morphine and clonidine combination therapy improves therapeutic window in mice: synergy in antinociceptive but not in sedative or cardiovascular effects. PLoS One 9: e109903 10.1371/journal.pone.0109903 25299457 PMC4192360

[12] Due MR, Yang XF, Allette YM, Randolph AL, Ripsch MS, Wilson SM, et al (2014) Carbamazepine potentiates the effectiveness of morphine in a rodent model of neuropathic pain. PLoS One 9: e107399 10.1371/journal.pone.0107399 25221944 PMC4164621

[13] Boroujerdi A, Zeng J, Sharp K, Kim D, Steward O, Luo ZD (2011) Calcium channel alpha-2-delta–1 protein upregulation in dorsal spinal cord mediates spinal cord injury-induced neuropathic pain states. Pain 152: 649–655. 21239111 10.1016/j.pain.2010.12.014 PMC3039050

[14] Thomas B, Farquhar-Smith P (2011) Extended-release gabapentin in post-herpetic neuralgia. Expert opinion on pharmacotherapy 12: 2565–2571. 10.1517/14656566.2011.622267 21942929

[15] Vonsy JL, Ghandehari J, Dickenson AH (2009) Differential analgesic effects of morphine and gabapentin on behavioural measures of pain and disability in a model of osteoarthritis pain in rats. Eur J Pain 13: 786–793. 10.1016/j.ejpain.2008.09.008 18955000

[16] Luo ZD, Chaplan SR, Higuera ES, Sorkin LS, Stauderman KA, Williams ME, et al (2001) Upregulation of dorsal root ganglion (alpha)2(delta) calcium channel subunit and its correlation with allodynia in spinal nerve-injured rats. J Neurosci 21: 1868–1875. 11245671 10.1523/JNEUROSCI.21-06-01868.2001 PMC6762626

[17] Kukkar A, Bali A, Singh N, Jaggi AS (2013) Implications and mechanism of action of gabapentin in neuropathic pain. Arch Pharm Res 36: 237–251. 10.1007/s12272-013-0057-y 23435945

[18] Meymandi MS, Sepehri G (2008) Gabapentin action and interaction on the antinociceptive effect of morphine on visceral pain in mice. Eur J Anaesthesiol 25: 129–134. 17697423 10.1017/S0265021507001226

[19] Bao YH, Zhou QH, Chen R, Xu H, Zeng LL, Zhang X, et al (2014) Gabapentin Enhances the Morphine Anti-Nociceptive Effect in Neuropathic Pain via the Interleukin-10-Heme Oxygenase–1 Signalling Pathway in Rats. J Mol Neurosci. 10.1007/s12031-014-0262-2 PMC4125805 24573601

[20] Singh S, Carpenter AE, Genovesio A (2014) Increasing the Content of High-Content Screening An Overview. Journal of biomolecular screening: 1087057114528537. 10.1177/1087057114528537 PMC4230961 24710339

[21] Charoenkwan P, Hwang E, Cutler RW, Lee H-C, Ko L-W, Huang HL, et al (2013) HCS-Neurons: identifying phenotypic changes in multi-neuron images upon drug treatments of high-content screening. BMC Bioinformatics 14: S12. 10.1186/1471-2105-14-S16-S12 PMC3853092 24564437

[22] Otsu N (1975) A threshold selection method from gray-level histograms. Automatica 11: 23–27.

[23] Hu M-K (1962) Visual pattern recognition by moment invariants. Information Theory, IRE Transactions on 8: 179–187.

[24] Teh C-H, Chin RT (1988) On image analysis by the methods of moments. Pattern Analysis and Machine Intelligence, IEEE Transactions on 10: 496–513.

[25] Mukundan R, Ong S, Lee PA (2001) Image analysis by Tchebichef moments. Image Processing, IEEE Transactions on 10: 1357–1364. 10.1109/83.941859 18255550

[26] Yap P-T, Paramesran R, Ong S-H (2003) Image analysis by Krawtchouk moments. Image Processing, IEEE Transactions on 12: 1367–1377. 10.1109/TIP.2003.818019 18244694

[27] Teague MR (1980) Image analysis via the general theory of moments*. JOSA 70: 920–930.

[28] Sheng Y, Duvernoy J (1986) Circular-Fourier-radial-Mellin transform descriptors for pattern recognition. JOSA A 3: 885–888. 10.1364/josaa.3.000885 3734928

[29] Haralick RM (1979) Statistical and structural approaches to texture. Proceedings of the IEEE 67: 786–804.

[30] Clausi DA (2002) An analysis of co-occurrence texture statistics as a function of grey level quantization. Canadian Journal of remote sensing 28: 45–62.

[31] Goshtasby A (1985) Description and discrimination of planar shapes using shape matrices. Pattern Analysis and Machine Intelligence, IEEE Transactions on: 738–743. 10.1109/tpami.1985.4767734 21869316

[32] Lu G, Sajjanhar A (1999) Region-based shape representation and similarity measure suitable for content-based image retrieval. Multimedia Systems 7: 165–174.

[33] Zhang D, Lu G (2004) Review of shape representation and description techniques. Pattern recognition 37: 1–19.

[34] Huang K, Murphy RF (2004) Boosting accuracy of automated classification of fluorescence microscope images for location proteomics. Bmc Bioinformatics 5: 78 15207009 10.1186/1471-2105-5-78 PMC449699

[35] Zhang D, Lu G (2002) Shape-based image retrieval using generic Fourier descriptor. Signal Processing: Image Communication 17: 825–848.

[36] Maragos P (1989) Pattern spectrum and multiscale shape representation. Pattern Analysis and Machine Intelligence, IEEE Transactions on 11: 701–716.

[37] Lee K-L, Chen L-H (2005) An efficient computation method for the texture browsing descriptor of MPEG–7. Image and Vision Computing 23: 479–489.

[38] Abramoff MD, Magelhaes P.J., Ram S.J. (2004) Image Processing with ImageJ. Biophotonics International 11: 36–42.

[39] Ho SY, Chao CY, Huang HL, Chiu TW, Charoenkwan P, Hwang E (2011) NeurphologyJ: An automatic neuronal morphology quantification method and its application in pharmacological discovery. Bmc Bioinformatics 12. 10.1186/1471-2105-12-230 PMC3121649 21651810

[40] Liu W, Wang CH, Cui Y, Mo LQ, Zhi JL, Sun SN, et al (2006) Inhibition of neuronal nitric oxide synthase antagonizes morphine antinociceptive tolerance by decreasing activation of p38 MAPK in the spinal microglia. Neurosci Lett 410: 174–177. 17101217 10.1016/j.neulet.2006.08.091

[41] Bekheet SH, Saker SA, Abdel-Kader AM, Younis AE (2010) Histopathological and biochemical changes of morphine sulphate administration on the cerebellum of albino rats. Tissue Cell 42: 165–175. 10.1016/j.tice.2010.03.005 20434749

[42] Ho S-Y, Chen J-H, Huang M-H (2004) Inheritable genetic algorithm for biobjective 0/1 combinatorial optimization problems and its applications. Systems, Man, and Cybernetics, Part B: Cybernetics, IEEE Transactions on 34: 609–620. 10.1109/tsmcb.2003.817090 15369097

[43] Ho S-Y, Shu L-S, Chen J-H (2004) Intelligent evolutionary algorithms for large parameter optimization problems. Evolutionary Computation, IEEE Transactions on 8: 522–541.

[44] Chang C-C, Lin C-J (2011) LIBSVM: a library for support vector machines. ACM Transactions on Intelligent Systems and Technology (TIST) 2: 27.

[45] Huang HL, Lin IC, Liou YF, Tsai CT, Hsu KT, Huang WL, et al (2011) Predicting and analyzing DNA-binding domains using a systematic approach to identifying a set of informative physicochemical and biochemical properties. BMC Bioinformatics 12 Suppl 1: S47 10.1186/1471-2105-12-S1-S47 21342579 PMC3044304

[46] Hutchinson MR, Zhang Y, Shridhar M, Evans JH, Buchanan MM, Zhao TX, et al (2010) Evidence that opioids may have toll-like receptor 4 and MD–2 effects. Brain Behav Immun 24: 83–95. 19679181 10.1016/j.bbi.2009.08.004 PMC2788078

[47] Kilic FS, Sirmagul B, Yildirim E, Oner S, Erol K (2012) Antinociceptive effects of gabapentin & its mechanism of action in experimental animal studies. Indian J Med Res 135: 630–635. 22771591 PMC3401692

[48] Yuan YM, He C (2013) The glial scar in spinal cord injury and repair. Neurosci Bull 29: 421–435. 10.1007/s12264-013-1358-3 23861090 PMC5561940

[49] Zhang X, Chen Y, Wang C, Huang L-Y (2007) Neuronal somatic ATP release triggers neuron—satellite glial cell communication in dorsal root ganglia. Proceedings of the National Academy of Sciences 104: 9864–9869. 10.1073/pnas.0611048104 PMC1887586 17525149

[50] Li Z, Fang ZY, Xiong L, Huang XL (2010) Spinal cord injury-induced astrocyte migration and glial scar formation: effects of magnetic stimulation frequency. Indian J Biochem Biophys 47: 359–363. 21355419

[51] Perry VH, Nicoll JA, Holmes C (2010) Microglia in neurodegenerative disease. Nat Rev Neurol 6: 193–201. 10.1038/nrneurol.2010.17 20234358

[52] Zou J, Vetreno RP, Crews FT (2012) ATP-P2X7 receptor signaling controls basal and TNFα‐stimulated glial cell proliferation. Glia 60: 661–673. 10.1002/glia.22302 22298391 PMC3276752

