# Supplementary material for: Predicting Neuroinflammation in Morphine Tolerance for Tolerance Therapy from Immunostaining Images of Rat Spinal Cord
Source: PLoS One. 2015 Oct 5;10(10):e0139806. doi: 10.1371/journal.pone.0139806 (PMC4593634; doi:10.1371/journal.pone.0139806)
Supplement: S1 Table — (DOCX) [file pone.0139806.s001.docx]

# Table S1. The 40 features of univariate feature selection for predicting images of morphine-tolerant astrocytes.

| Features Name | Feature type | Feature type |
| --- | --- | --- |
| 1. Shape Distribution X/Y (2,0) | BW_Geometric_2_0 | Interpretable |
| 1. Shape Distribution X/Y (0,2) | BW_Geometric_0_2 | Interpretable |
| 1. Shape Solidity | BW_Solidity, the solidity of visible pixel area. | Interpretable |
| 1. Pixel Area (1) | Histogram_1, the number of pixel in specific range of intensity. | Interpretable |
| 1. Pixel Area (10) | Histogram_10, the number of pixel in specific range of intensity. | Interpretable |
| 1. Pixel Area (11) | Histogram_11, the number of pixel in specific range of intensity. | Interpretable |
| 1. Pixel Area (12) | Histogram_12, the number of pixel in specific range of intensity. | Interpretable |
| 1. Pixel Area (13) | Histogram_13, the number of pixel in specific range of intensity. | Interpretable |
| 1. Pixel Area Ring (121) | BW_Ring_R121, pixel area close to the center of image. | Interpretable |
| 1. Texture Area (1,1,1) | GLCM_MEAN_D1R1C1, the occurrences of pairs of pixels that satisfy specific condition. | Interpretable |
| 1. Texture Area (3,1,1) | GLCM_MEAN_D3R1C1, the occurrences of pairs of pixels that satisfy specific condition. | Interpretable |
| 1. Texture Area (3,5,3) | GLCM_MEAN_D3R5C3, the occurrences of pairs of pixels that satisfy specific condition. | Interpretable |
| 1. Texture Area (5,4,2) | GLCM_MEAN_D5R4C2, the occurrences of pairs of pixels that satisfy specific condition. | Interpretable |
| 1. Texture consistency (1) | MEAN_Harr_Energy_D1, the consistency of image intensities that satisfy specific condition. | Interpretable |
| 1. Texture consistency (3) | MEAN_Harr_Energy_D3, the consistency of image intensities that satisfy specific condition. | Interpretable |
| 1. Texture consistency (5) | MEAN_Harr_Energy_D5, the consistency of image intensities that satisfy specific condition. | Interpretable |
| 1. Texture solidity (1) | MEAN_Harr_MaxProbability_D1, the number of most occurrences of pairs of pixels intensities that satisfy specific condition. | Interpretable |
| 1. Texture solidity (3) | MEAN_Harr_MaxProbability_D3, the number of most occurrences of pairs of pixels intensities that satisfy specific condition. | Interpretable |
| 1. Texture randomness (1) | MEAN_Harr_SumEntropy_D1, the randomness of pairs of pixels that satisfy specific condition. | Interpretable |
| 1. Texture randomness (3) | MEAN_Harr_SumEntropy_D3, the randomness of pairs of pixels that satisfy specific condition. | Interpretable |
| 1. Texture randomness (5) | MEAN_Harr_SumEntropy_D5, the randomness of pairs of pixels that satisfy specific condition. | Interpretable |
| 1. Texture randomness2 (1) | MEAN_Harr_Entropy_D1, the randomness of pairs of pixels that satisfy specific condition. | Interpretable |
| 1. Texture randomness2 (3) | MEAN_Harr_Entropy_D3, the randomness of pairs of pixels that satisfy specific condition. | Interpretable |
| 1. Texture randomness2 (5) | MEAN_Harr_Entropy_D5, the randomness of pairs of pixels that satisfy specific condition. | Interpretable |
| 1. Texture randomness variation (3) | STD_Harr_Entropy_D3, the variation of randomness in four directions of pairs of pixels that satisfy specific condition. | Interpretable |
| 1. Texture randomness variation (5) | STD_Harr_Entropy_D5, the variation of randomness in four directions of pairs of pixels that satisfy specific condition. | Interpretable |
| 1. Texture randomness variation2 (3) | STD_Harr_DifEntropy_D3, the variation of randomness in four directions of pairs of pixels that satisfy specific condition. | Interpretable |
| 1. Texture information (1) | MEAN_Harr_InfoCorrelation2_D1, the information measure of intensity values correlation that satisfies specific condition. | Interpretable |
| 1. Texture information (3) | MEAN_Harr_InfoCorrelation2_D3, the information measure of intensity value correlation that satisfies specific condition. | Interpretable |
| 1. Zernike (Binary, 2,0) | Polar-coordinate-based moments | Computational |
| 1. Pseudo Zernike (Binary, 1,0) | Polar-coordinate-based moments | Computational |
| 1. Fourier Mellin (Binary, 1,0) | Polar-coordinate-based moments | Computational |
| 1. Radial Tchebichef Fourier (Binary, 0,0) | Polar-coordinate-based moments | Computational |
| 1. Legendre (Binary, 2,0) | Cartesian-coordinate-based moments | Computational |
| 1. Tchebichef (Binary, 2,0) | Cartesian-coordinate-based moments | Computational |
| 1. Mean Cell Intensity | Mean Intensity | Neuronal |
| 1. Cell Number | Object Number | Neuronal |
| 1. Total Cell Area | Total Object Area | Neuronal |
| 1. Large Cell Area | Large Object Area | Neuronal |
| 1. Large Cell Diameter | Large Object Equivalent Diameter | Neuronal |
