# Supplementary material for: Predicting Neuroinflammation in Morphine Tolerance for Tolerance Therapy from Immunostaining Images of Rat Spinal Cord
Source: PLoS One. 2015 Oct 5;10(10):e0139806. doi: 10.1371/journal.pone.0139806 (PMC4593634; doi:10.1371/journal.pone.0139806)
Supplement: S2 Table — (DOCX) [file pone.0139806.s002.docx]

# Table S2. The 40 features of univariate feature selection for predicting images of morphine-tolerant microglia.

| Features Name | Type of Description | Feature type |
| --- | --- | --- |
| 1. Shape Distribution X/Y (2,0) | Gray_Geometric_2_0 | Interpretable |
| 1. Texture Area Variation (1,5,3) | GLCM_STD_D1R5C3, the variation of occurrences of pairs of pixels in four directions that satisfy specific condition | Interpretable |
| 1. Texture Area Variation (1,5,5) | GLCM_STD_D1R5C5, the variation of occurrences of pairs of pixels in four directions that satisfy specific condition | Interpretable |
| 1. Texture Area Variation (3,2,6) | GLCM_STD_D3R2C6, the variation of occurrences of pairs of pixels in four directions that satisfy specific condition | Interpretable |
| 1. Texture Area Variation (3,5,4) | GLCM_STD_D3R5C4, the variation of occurrences of pairs of pixels in four directions that satisfy specific condition. | Interpretable |
| 1. Texture Area Variation (3,5,6) | GLCM_STD_D3R5C6, the variation of occurrences of pairs of pixels in four directions that satisfy specific condition | Interpretable |
| 1. Texture Area Variation (3,5,7) | GLCM_STD_D3R5C7, the variation of occurrences of pairs of pixels in four directions that satisfy specific condition | Interpretable |
| 1. Texture Area Variation (5,4,1) | GLCM_STD_D5R4C1, the variation of occurrences of pairs of pixels in four directions that satisfy specific condition. | Interpretable |
| 1. Texture Area Variation (5,5,4) | GLCM_STD_D5R5C4, the variation of occurrences of pairs of pixels in four directions that satisfy specific condition. | Interpretable |
| 1. Texture Area Variation (5,7,3) | GLCM_STD_D5R7C3, the variation of occurrences of pairs of pixels in four directions that satisfy specific condition | Interpretable |
| 1. Pixel Intensity Variation (Gabor, 1,5) | Gabor_STD_S1O5, the variation of pixel intensity of Gabor-filtered image | Interpretable |
| 1. Pixel Intensity Variation (Gabor, 1,6) | Gabor_STD_S1O6, the variation of pixel intensity of Gabor-filtered image | Interpretable |
| 1. Pixel Intensity Variation (Gabor, 2,5) | Gabor_STD_S2O5, the variation of pixel intensity of Gabor-filtered image | Interpretable |
| 1. Pixel Intensity Variation (Gabor, 3,5) | Gabor_STD_S3O5, the variation of pixel intensity of Gabor-filtered image | Interpretable |
| 1. Pixel Intensity Variation (Gabor, 4,5) | Gabor_STD_S4O5, the variation of pixel intensity of Gabor-filtered image | Interpretable |
| 1. Legendre (Binary, 0,4) | Cartesian-coordinate-based moments | Computational |
| 1. Legendre (Binary, 2,4) | Cartesian-coordinate-based moments | Computational |
| 1. Legendre (Gray, 0,2) | Cartesian-coordinate-based moments | Computational |
| 1. Legendre (Gray, 2,4) | Cartesian-coordinate-based moments | Computational |
| 1. Tchebichef (Binary, 0,4) | Cartesian-coordinate-based moments | Computational |
| 1. Tchebichef (Binary, 2,4) | Cartesian-coordinate-based moments | Computational |
| 1. Tchebichef (Gray, 0,2) | Cartesian-coordinate-based moments | Computational |
| 1. Tchebichef (Gray, 0,4) | Cartesian-coordinate-based moments | Computational |
| 1. Tchebichef (Gray, 2,4) | Cartesian-coordinate-based moments | Computational |
| 1. Krawtchouk (Binary, 11,16) | Cartesian-coordinate-based moments | Computational |
| 1. Fourier Mellin (Binary, 13,12) | Polar-coordinate-based moments | Computational |
| 1. Fourier Mellin (Gray, 12,6) | Polar-coordinate-based moments | Computational |
| 1. Fourier Mellin (Gray, 16,7) | Polar-coordinate-based moments | Computational |
| 1. Pseudo Zernike (Binary, 15,5) | Polar-coordinate-based moments | Computational |
| 1. Fourier (Mean, Ring,1.1719) | Power spectrum of Fourier transform | Computational |
| 1. Fourier (Mean, Ring,9.375) | Power spectrum of Fourier transform | Computational |
| 1. Fourier (Mean, Ring, 1.1719, Wedges, -45) | Power spectrum of Fourier transform | Computational |
| 1. Fourier (Mean, Ring, 9.375, Wedges, 45) | Power spectrum of Fourier transform | Computational |
| 1. Wavelet (db4, Diagonal,5) | Wavelet energy | Computational |
| 1. Wavelet (haar, Diagonal,5) | Wavelet energy | Computational |
| 1. Mean Cell Intensity | Mean Intensity | Neuronal |
| 1. Cell Number | Object Number | Neuronal |
| 1. Total Cell Area | Total Object Area | Neuronal |
| 1. Large Cell Area | Large Object Area | Neuronal |
| 1. Large Cell Diameter | Large Object Equivalent Diameter | Neuronal |
