# Supplementary material for: Predicting Neuroinflammation in Morphine Tolerance for Tolerance Therapy from Immunostaining Images of Rat Spinal Cord
Source: PLoS One. 2015 Oct 5;10(10):e0139806. doi: 10.1371/journal.pone.0139806 (PMC4593634; doi:10.1371/journal.pone.0139806)
Supplement: S3 Table — (DOCX) [file pone.0139806.s003.docx]

# Table S3. The 40 features of univariate feature selection for predicting images of morphine-tolerant neurons.

| Features Name | Type of Description | Feature type |
| --- | --- | --- |
| 1. Shape Distribution X/Y (0,4) | BW_Geometric_0_4 | Interpretable |
| 1. Shape Distribution X/Y (0,6) | BW_Geometric_0_6 | Interpretable |
| 1. Different Pixel Area (30) | Relative_Histogram_30, the different between percentage of pixel in 29^th^ bin and 30^th^ bin of histogram | Interpretable |
| 1. Pixel Area Ring Wedges (361,162) | BW_Ring_R361W162, the percentage of pixel in specific area defined by ring and wedge | Interpretable |
| 1. Legendre (Binary, 1,15) | Cartesian-coordinate-based moments | Computational |
| 1. Legendre (Binary, 15,5) | Cartesian-coordinate-based moments | Computational |
| 1. Legendre (Gray, 1,15) | Cartesian-coordinate-based moments | Computational |
| 1. Legendre (Gray, 10,12) | Cartesian-coordinate-based moments | Computational |
| 1. Legendre (Gray, 15,5) | Cartesian-coordinate-based moments | Computational |
| 1. Legendre (Gray, 16,3) | Cartesian-coordinate-based moments | Computational |
| 1. Tchebichef (Binary, 1,15) | Cartesian-coordinate-based moments | Computational |
| 1. Tchebichef (Binary, 15,5) | Cartesian-coordinate-based moments | Computational |
| 1. Tchebichef (Gray, 1,15) | Cartesian-coordinate-based moments | Computational |
| 1. Tchebichef (Gray, 10,12) | Cartesian-coordinate-based moments | Computational |
| 1. Tchebichef (Gray, 15,5) | Cartesian-coordinate-based moments | Computational |
| 1. Tchebichef (Gray, 16,3) | Cartesian-coordinate-based moments | Computational |
| 1. Krawtchouk (Gray, 1,5) | Cartesian-coordinate-based moments | Computational |
| 1. Krawtchouk (Gray, 4,13) | Cartesian-coordinate-based moments | Computational |
| 1. Radial Tchebichef-Fourier (Binary, 2,1) | Polar-coordinate-based moments | Computational |
| 1. Radial Tchebichef-Fourier (Binary, 2,7) | Polar-coordinate-based moments | Computational |
| 1. Radial Tchebichef-Fourier (Binary, 3,4) | Polar-coordinate-based moments | Computational |
| 1. Radial Tchebichef-Fourier (Binary, 7,12) | Polar-coordinate-based moments | Computational |
| 1. Radial Tchebichef-Fourier (Binary, 13,8) | Polar-coordinate-based moments | Computational |
| 1. Radial Tchebichef-Fourier (Binary, 13,16) | Polar-coordinate-based moments | Computational |
| 1. Radial Tchebichef-Fourier (Gray, 0,6) | Polar-coordinate-based moments | Computational |
| 1. Radial Tchebichef-Fourier (Gray, 0,15) | Polar-coordinate-based moments | Computational |
| 1. Fourier Mellin (Binary, 6,6) | Polar-coordinate-based moments | Computational |
| 1. Fourier Mellin (Binary, 15,10) | Polar-coordinate-based moments | Computational |
| 1. Fourier Mellin (Binary, 16,1) | Polar-coordinate-based moments | Computational |
| 1. Fourier Mellin (Gray, 0,15) | Polar-coordinate-based moments | Computational |
| 1. Fourier Mellin (Gray, 7,10) | Polar-coordinate-based moments | Computational |
| 32. Fourier Mellin (Gray, 8,10) | Polar-coordinate-based moments | Computational |
| 1. Zernike (Binary, 14,4) | Polar-coordinate-based moments | Computational |
| 1. Pseudo Zernike (Binary, 8,4) | Polar-coordinate-based moments | Computational |
| 1. Pseudo Zernike (Binary, 16,1) | Polar-coordinate-based moments | Computational |
| 36. Mean Cell Intensity | Mean Intensity | Neuronal |
| 37. Cell Number | Object Number | Neuronal |
| 38. Total Cell Area | Total Object Area | Neuronal |
| 39. Large Cell Area | Large Object Area | Neuronal |
| 40. Large Cell Diameter | Large Object Equivalent Diameter | Neuronal |
